# Species competition: coexistence, exclusion and clustering

**DOI:** 10.1098/rsta.2009.0086

**Published:** 2009-08-28

**Authors:** Emilio Hernández-García, Cristóbal López, Simone Pigolotti, Ken H. Andersen

**Affiliations:** 1 IFISC (UIB-CSIC), Instituto de Física Interdisciplinar y Sistemas Complejos, Campus Universitat de les Illes Balears, 07122 Palma de Mallorca, Spain; 2 The Niels Bohr Institute, The Niels Bohr International Academy, Blegdamsvej 17, 2100, Copenhagen, Denmark; 3 National Institute of Aquatic Resources, Technical University of Denmark, Charlottenlund Slot, 2920 Charlottenlund, Denmark

**Keywords:** competition, Lotka–Volterra, competitive exclusion, limiting similarity, pattern formation

## Abstract

We present properties of Lotka–Volterra equations describing ecological competition among a large number of interacting species. First we extend previous stability conditions to the case of a non-homogeneous niche space, i.e. that of a carrying capacity depending on the species trait. Second, we discuss mechanisms leading to species clustering and obtain an analytical solution for a state with a lumped species distribution for a specific instance of the system. We also discuss how realistic ecological interactions may result in different types of competition coefficients.

## 1. Lotka–Volterra competition and species distribution

Competitive interactions occur when entities in a system grow by consuming common finite resources. They are ubiquitous in many fields of science: examples include biological species competing for food (MacArthur & Levins 1967; Roughgarden 1979; Case 1981), mode competition in nonlinear optical systems (Benkert & Anderson 1991), or alternative technologies competing for a market (Pistorius & Utterback 1997). An early, simple, but powerful model for competitive interactions is the Lotka–Volterra (LV) set of competition equations (Volterra 1926; Lotka 1932),
1.1
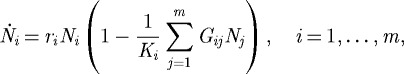

where *m* is the number of species, *N*_*i*_ is the population of species *i*, *r*_*i*_ is its maximum growth rate, *K*_*i*_ is its carrying capacity and *G*_*ij*_ is the matrix characterizing the interaction among species *i* and *j*, more specifically the decrease in the growth rate of species *i* due to the presence of *j*. Competitive interactions are characterized by *G*_*ij*_≥0, the situation to be considered here, whereas negative interactions may model situations of mutualism, predation or symbiosis.

In classical ecological niche theory, species are associated to points in an abstract niche space. Coordinates in this space represent relevant phenotypic characteristics, for example size of individuals in a species, or the size of preferred prey, such that intensity of competition is larger if species are closer in this space. For simplicity we assume this space to be one-dimensional (multi-dimensional generalizations are straightforward, as briefly mentioned later). If niche locations can be considered to be a continuum, we can write equation (1.1) as
1.2
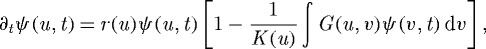

where *ψ*(*u*,*t*) is the population density at niche location *u*. The integral extends over the full niche space, which could be finite or infinite. For most purposes, equations (1.1) and (1.2) can be considered as equivalent, since the second is obtained from the first in the limit of many close interacting species, and equation (1.1) can be recovered from equation (1.2) for a discrete distribution of species,
1.3
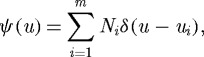

with *G*_*ij*_=*G*(*u*_*i*_,*u*_*j*_), *r*_*i*_=*r*(*u*_*i*_) and *K*_*i*_=*K*(*u*_*i*_).

It is widely believed that equations (1.1) or (1.2) predict a competitive exclusion leading to a limiting similarity situation (Abrams 1983), in which a pair of species too close in niche space cannot coexist, and one of them would become extinct. However, it is known that the model allows for continuous coexistence of species in some situations (Roughgarden 1979) and refinements on the conditions for this coexistence have been developed with emphasis on the effect of the shape of the carrying capacity function *K*(*u*) (Meszéna *et al.* 2006; Szabó & Meszéna 2006). In this context, a particularly surprising result was the finding by Scheffer & van Nes (2006) of a situation—for uniform carrying capacity—which was neither of full coexistence nor of full exclusion, but of clusters or lumps of tightly packed species which did not exclude each other, but were well separated from other clusters so that there was a type of limiting similarity leading to a minimum intercluster distance. Clustering of individuals or entities under competitive interactions of the LV type had already been observed in other contexts (Fuentes *et al.* 2003; Hernández-García & López 2004, 2005; Ramos *et al.* 2008), where the mechanism was the diffusive broadening of an otherwise zero-width species or entity. In contrast, the lumps in Scheffer & van Nes (2006) appeared even in the absence of diffusion in niche space, which is the situation also considered here.

The importance of the functional form of the interaction kernel *G*_*ij*_ in equation (1.1) or *G*(*u*,*v*) in equation (1.2) was stressed by Pigolotti *et al.* (2007) for the case of uniform carrying capacity and interactions depending only on differences of niche positions, and found to be relevant in an evolutionary context by Leimar *et al.* (2008). For that case the positive-definite character of the Fourier transform of *G*(*u*,*v*)=*G*(*u*−*v*) is a condition implying the absence of limiting similarity. Species clustering was reported, but for interaction functions rather different from the Gaussian used in Scheffer & van Nes (2006). In fact, for the Gaussian interaction case most results are extremely sensitive to details such as the implementation of the boundary conditions or weak ecological second-order effects (Pigolotti *et al.* 2008). Thus, a clarification of the mechanisms leading to species clustering in LV models would be desirable. In addition, the results in Pigolotti *et al.* (2007, 2008) were obtained under the unrealistic assumption of homogeneity in niche space, whereas the inhomogeneities in the carrying capacity are known to play relevant roles (Szabó & Meszéna 2006). For simplicity we restrict our description to the standard situation in which competition is stronger among species closer in niche space. The existence of studies of LV systems where non-local interactions beyond that type are considered is worth mentioning (Doebeli & Dieckmann 2000). That situation can also be described by the general formalism used here of an integral kernel function, and our general results therefore also apply to the situation with more general non-local interactions.

In this paper we analyse some mathematical properties of the LV model (1.1) or (1.2). In §2 we show that the positive-definiteness of the kernel *G* remains a determining condition for stable coexistence even for non-constant *K*(*u*). In §3, we discuss the mechanism producing lumped species distributions and explicitly provide an analytical expression for a particular interaction kernel. In the appendix we show that, in contrast with the earliest characterizations of the interaction kernel *G* (MacArthur & Levins 1967; Roughgarden 1979), both positive- and non-positive-definite kernels can arise from more detailed ecological models which consider the dynamics of the consumed resource. We use periodic boundary conditions in our numerical simulations. We expect the effects of this simplifying but unrealistic assumption to be unimportant at least when a non-constant carrying capacity limits the presence of species to a limited region of niche space.

## 2. The stability of close coexistence

A simplifying assumption for the study of the LV model is that of homogeneity in niche space. In this case, the carrying capacity and growth rate are constants, *K*_0_ and *r*_0_, and the interaction kernel depends only on differences of niche positions *G*(*u*,*v*)=*G*(|*u*−*v*|). Niche space could be infinite, but, in the case in which it is finite, homogeneity can only be achieved under periodic boundary conditions. Under these restrictions it is easy to see that a steady solution to equation (1.2) which is homogeneous and everywhere non-vanishing always exists: 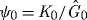, where 
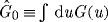
. This solution represents coexistence of all possible species without a limit to their similarity. Its stability against small perturbations can be analysed by linearization of the equation resulting from substitution of *ψ*(*u*,*t*)=*ψ*_0_+*δψ*(*u*,*t*) in equation (1.2). The solution for the Fourier transform of the deviation from the homogeneous state, 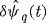, is
2.1
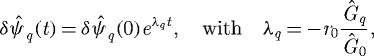

where 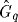 is the Fourier transform of *G*(*u*). Thus, the homogeneous solution *ψ*_0_ is stable if 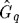 is positive ∀*q*, while a instability leading to pattern formation occurs when 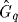 may take negative values (Fuentes *et al.* 2004; Hernández-García & López 2004; López & Hernández-García 2004; Pigolotti *et al.* 2007). We note that many steady solutions to equation (1.2) exist besides *ψ*_0_ (in particular, solutions of the form equation (1.3)). This is so because dynamics preserves *ψ*(*u*)=0 at all places where there is no initial population. Notice also that *ψ*_0_ is the only strictly positive solution. Among this multiplicity of solutions the ones that will be more relevant are those which are stable under perturbations or small immigration (Pigolotti *et al.* 2007).

An interesting class of functions to be used as kernels and carrying capacities is the family 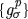 given by
2.2
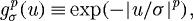

which is parameterized by the value of *p*. The widely used Gaussian kernel is obtained for *p*=2. When *p*<2 the functions are more peaked around *u*=0 (the case *p*=1 is an exponential) and for *p*>2 they become more box-like (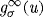 is the flat box with value 1 in the interval [−*σ*,*σ*] and zero outside). The width of the kernel *σ* gives the competition range in niche space. We have positivity of the Fourier transform if *p*≤2. This implies that the homogeneous solution is stable under evolution with uniform *K* and kernel *G* of the form equation (2.2) if *p*≤2. When *p*>2, the homogeneous solution is unstable and the system approaches delta comb solutions of the type equation (1.3), with a spacing approximately 1.4*σ* (Pigolotti *et al.* 2007) that represents limiting similarity situations.

We now generalize the above stability analysis to the more realistic case in which there is no homogeneity in niche space. First, we consider the simpler case of a symmetric kernel *G*(*u*,*v*)=*G*(*v*,*u*), which in particular includes the previous case of kernels depending only on species distance: *G*(*u*,*v*)=*G*(|*u*−*v*|). Note that in this symmetric case one can write equation (1.2) in potential form,
2.3
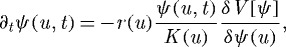

with the functional potential given by
2.4
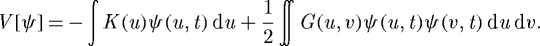



Stationary solutions of equation (1.2) are those for which the right-hand side of equation (2.3) equals 0. This has many possible solutions. We define the *natural stationary solution*, *ψ*^*N*^(*u*), as the one which is positive and non-vanishing for all *u*, so that
2.5
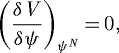

that is, the one satisfying
2.6
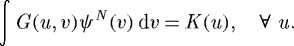

The solution *ψ*^*N*^(*u*) can be considered as the non-homogeneous generalization of *ψ*_0_ introduced in the homogeneous case. In the particular case in which *G*(*u*,*v*)=*G*(*u*−*v*), the natural solution can be explicitly written in terms of Fourier transforms of the competition kernel and the carrying capacity, either in an infinite system or in a finite one with periodic boundary conditions,
2.7
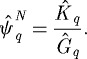

This requires that these Fourier transforms and their inverses exist and lead to positive population densities. When this happens, a continuum species coexistence is obtained, and its existence is generally robust against small changes in *G* or *K*. Later we show that it is also an attractor of the dynamics when 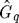 satisfy positivity requirements (*p*≤2, for the family in equation (2.2), being *p*=2 the marginal case). For a uniform carrying capacity, the natural solution equation (2.7) always exists and is uniform in phenotype space *ψ*^*N*^(*u*)=*ψ*_0_. But the natural solution may lose positivity or even cease to exist depending on the properties of *G* and *K*. For example, when both *G*(*u*) and *K*(*u*) are of the form equation (2.2) with *p*=2, the inverse Fourier transform of equation (2.7) exists when the carrying capacity has a value of *σ* larger than the kernel *G*, but not in the opposite case.

**Figure 1.**
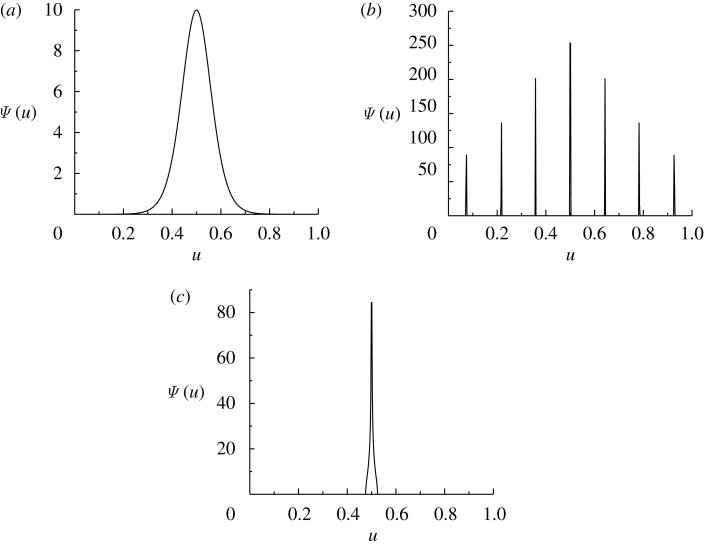
Long-time solutions of equation (1.2) for different kernels and carrying capacities. (*a*) 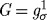, *K*=sech(*u*/*σ*), with *σ*=0.1. The natural steady solution (*ψ*^*N*^= *a*^−1^sech^3^(*u*/*σ*)), which is positive and non-vanishing everywhere, is reached at long times. (*b*) 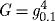, 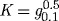. Under this non-positive-definite competition kernel, the solution shown is still evolving and approaches a singular delta comb of the type (1.3) at long times. (*c*) 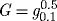, 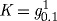. A positive natural solution does not exist and the system approaches a single hump solution which vanishes in part of the niche space.

Figure 1 shows stationary solutions attained at long times by the dynamics in equation (1.2) illustrating the situations described above, starting from a smooth non-vanishing initial condition. In the first case we choose a kernel and carrying capacity functions (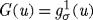, *K*(*u*)=*sech*(*u*/*σ*)), such that the natural solution exists and is positive everywhere. Thus it is stable, and it is the steady state attained at long times. In fact it can be analytically calculated,
2.8
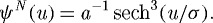



In the second case the non-positiveness of the kernel used (with a carrying capacity of the type equation (2.2)) breaks down the initial configuration into lumps, which at long times approach zero-width delta functions with forbidden zones in between. In the third case, despite 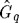 being positive, a positive natural solution does not exist. Several outcomes are possible but, for the kernel and capacity used, the system approaches a single hump solution which vanishes in part of the niche space.

More in general, but still in the symmetric *G* case *G*(*u*,*v*)=*G*(*v*,*u*), writing the LV model in potential form (equation (2.3)) is of great use since one can show that, provided *r*(*u*) and *K*(*u*) are positive, d*V*/d*t*≤0. This implies that *V* is a Lyapunov potential and dynamics proceeds towards its absolute minimum, or if *ψ*(*u*,*t*=0)=0 for some *u*, towards the minimum of *V* under such constrain. Notice that, since the potential is a quadratic form, *ψ*^*N*^ is a *global* attractor (starting from non-vanishing initial conditions) when the competition kernel is a positive-definite quadratic form, which means that 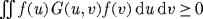, ∀*f* (or 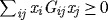, ∀{*x*_*i*_} in the discrete case). This generalizes the previous stability condition on the Fourier transform 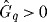 to niche inhomogeneous cases, and shows that the stability result was global indeed. In a multi-dimensional niche space, the same analysis shows that the positive-definiteness of the quadratic form remains the condition for the global stability of the natural solution. In any case, the important consequence is that the stability of the natural solution depends uniquely on the competition kernel and not on the carrying capacity (provided the relation kernel–capacity is such that the natural solution exists and is positive). In particular, for competition kernels of the form equation (2.2), *ψ*^*N*^ is always (if existing and positive) a globally stable solution of the dynamics for *p*≤2, and unstable otherwise.

The crucial difference in the case of a non-symmetric competition kernel is that there is no obvious Lyapunov potential for the system. This implies that there are no global stability results available. However, local stability can be investigated. Let us consider a small perturbation of the positive natural solution *ψ*^*N*^(*u*)+*δψ*(*u*,*t*). To linear order, the perturbation evolves as
2.9
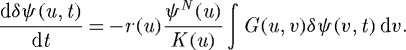

We now consider the functional 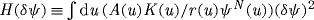, where *A*(*u*) is a positive function so that *H*≥0 and *H*(0)=0. Let us compute its time derivative,
2.10
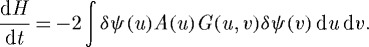



If for some choice of *A*(*u*) one has that *A*(*u*)*G*(*u*,*v*) is positive definite, then d*H*/d*t*<0 and *δψ*=0 will be approached. This shows that *ψ*^*N*^ is linearly stable in such a case. We note that the case in which *G*(*u*,*v*) itself is positive definite trivially guarantees the positivity of *A*(*u*)*G*(*u*,*v*), with a constant *A*. Thus, even in this more general non-symmetric case, it is the character of the interaction kernel *G*, and not of the carrying capacity (provided it is such that the natural solution exists and is positive), which determines the stability of the natural solution.

## 3. Lumped species distributions

**Figure 2.**
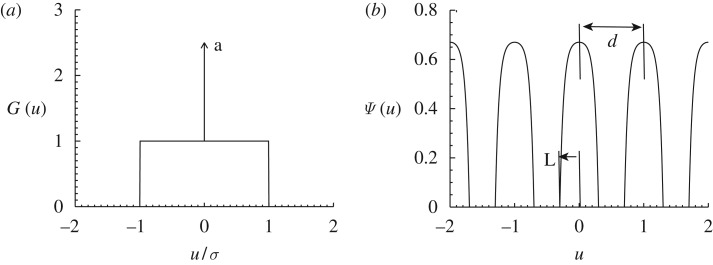
The kernel in equation (3.1) (*a*), and the analytical steady solution given by equations (3.5) and (3.8–3.9) for *a*=*K*_0_=1, *σ*=0.8, *L*=0.3 and *d*=1 (*b*).

Scheffer & van Nes (2006) found transient but long-lived solutions of equation (1.1) consisting of periodically spaced lumps containing many close species. They used a Gaussian interaction kernel which turned out to introduce an excessive sensitivity of the results to the numerical implementation of the model and boundary conditions (Pigolotti *et al.* 2008). However, they found similar solutions as steady configurations when adding an extra predation term acting effectively only on species with high population. This can be thought of as an extra *intraspecific* competition since it decreases the growth of species with many individuals. Exploiting this idea, Pigolotti *et al.* (2007) checked the effect of using in equation (1.2) a kernel of the type equation (2.2) but with an enhanced interaction at *u*=0, i.e. enhanced intraspecific competition. In particular, they used a constant carrying capacity *K*(*u*)=*K*_0_ and a flat box kernel with an added delta function at the origin (figure 2),
3.1
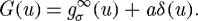

Lumped patterns were obtained numerically for *a*=1.

Because the dynamics of equation (1.2) usually involves very long transients, it is interesting to calculate analytically the steady lumped solution in the simple case of a kernel (3.1) and uniform carrying capacity *K*_0_ (in the infinite line).

We begin with the steady-state condition
3.2
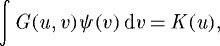

valid at *u* such that *ψ*(*u*)≠0, that particularized to equation (3.1) and constant *K* reads
3.3
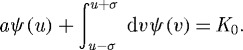

This is transformed into a differential-difference equation after differentiation with respect to *u*,
3.4
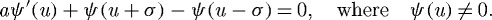

This steady equation has many solutions, including the natural one *ψ*_0_=*K*_0_/(*a*+2*σ*) which is non-vanishing everywhere, or delta combs such as equation (1.3). We search for solutions of the type in figure 2, i.e. periodic arrays of lumps, of period *d*, each one having a symmetric *hump* shape *f*(*u*) of width 2*L* (i.e. *f*(*u*)=0 if *u*∉[−*L*,*L*]),
3.5
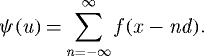

We are assuming that the lumps do not overlap, so that *d*>2*L*. We also note that if *σ*+2*L*<*d* there is no interaction between different lumps, so that for *u*∈[−*L*,*L*] equation (3.4) reduces to *f*′(*u*)=0 and there is no lump solution. Moreover, analysis is much simplified if each of the lumps interacts only with its neighbours (*σ*+2*L*<2*d*). Thus we restrict to *d*<*σ*+2*L*<2*d*, for which equations (3.4) with (3.5) and *u*∈(−*L*,*L*) becomes
3.6
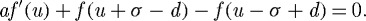

The general solution of this linear equation is obtained as a sum of exponentials 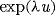, with
3.7
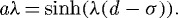

**Figure 3.**
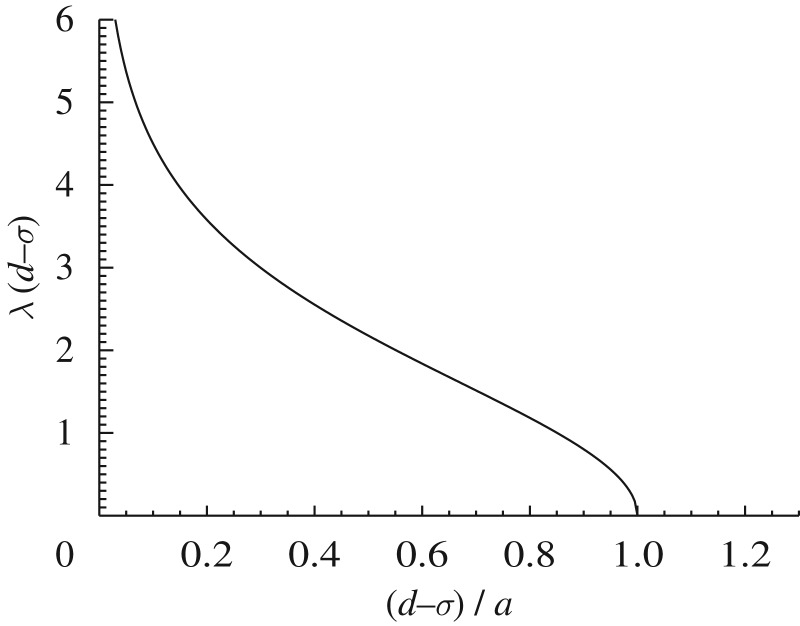
The solution *λ* (positive branch) of equation (3.7) giving the inverse width of species lumps. The width is finite for *d*−*σ*<*a*, which is favoured by larger enhanced intraspecific competition *a*.

*λ*=0 is always a solution, and if *d*−*σ*<*a* there are two additional solutions ±*λ*, plotted in figure 3. For *d*−*σ*>*a* the only solution is the constant one, but in the opposite case (the situation favoured by enhanced intraspecific competition *a*) the solution is a linear combination of three exponentials. Two of the constants of the combination are determined from *f*(*L*)=*f*(−*L*)=0. The third one, which gives the overall normalization, can be obtained by returning back to the original equation (3.3). Finally we get
3.8
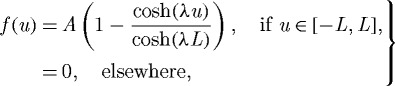

with
3.9
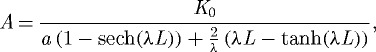

and the value of *λ* which is plotted in figure 3. Figure 2 shows the analytical solution equation (3.5) with equations (3.8) and (3.9). We have not studied the stability of this configuration. But the numerical results in Pigolotti *et al.* (2007) indicate that it is stable for some values of *L* and *d*.

**Figure 4.**
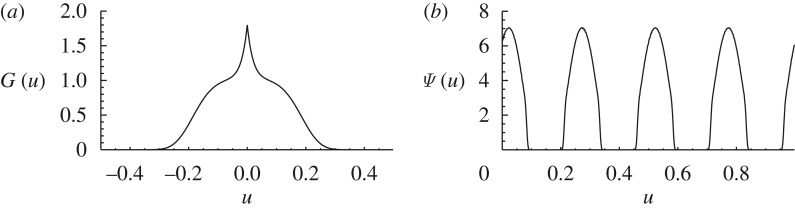
The kernel 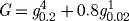 (*a*), and the steady solution obtained numerically from it at long times with constant *K*_0_=1 (*b*).

We finally stress that the appearance of the lumped solution is not a consequence of the singularity of the delta function in the kernel. In fact, any kernel sufficiently peaked at the origin will favour the coexistence of close species. If the behaviour at larger distances of the kernel makes it not positive definite, then full coexistence will be unstable and the natural solution will split into disjoint lumps. An example of the final steady state in this situation is shown in figure 4, with a kernel 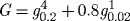, which has the properties just described and contains no delta singularity.

## Appendix A. Models leading to LV competitive interactions

We have seen that the character of the interaction kernel *G* is of major importance to determine the qualitative outcome of LV competition. In the original formulation of the niche model, however, only positive-definite kernels were allowed. The reason is that competition kernels were derived in terms of utilization functions *u*_*i*_(*x*), describing how consumer *i* uses resource at niche location *x* (assumed to be continuous) (MacArthur & Levins 1967; Roughgarden 1979),

A 1

When the resource is directly related to space, equation (1.1) can be justified by considering the probability that consumer *i* meets consumer *j* (Roughgarden 1979). It is easy to see that *G*_*i**j*_ obtained from (1.1) is positive definite. We show in the following, however, that relation (1.1) is by no means general, and that a greater variety of kernels—positive or non-positive definite, so that the natural solution representing coexistence can be either stable or unstable—could be obtained from equations in which resources are explicitly modelled. Related calculations could be found, for example, in Schoener (1974).

We consider a set of predators (or consumers) with populations *N*_*i*_, *i*=1,2,…,*m*, competing for different types of prey populations or resources, *R*_*α*_, *α*=1,2,…,*n*, the latter growing in a logistic way with growth rate *β*_*α*_ and carrying capacity *Q*_*α*_ in the absence of predators. Particular equations modelling this are

A 2

A 3

where *d*_*i*_ is the death rate of species *i*. The interaction coefficients are *a*_*α**i*_, the depletion rate of resource *α* produced by species *i*, and the sensitivity *S*_*i**α*_, giving the growth rate of *i* thanks to resource *α*. LV-type dynamics arises when the time scale for resource evolution is much faster than that of the consumers (i.e. *S*_*i**α*_ and , but with their ratio finite). In this case, adiabatic elimination of the resource can be done (, so that each prey is at each instant at the equilibrium determined by their consumers), giving

A 4

for the non-vanishing resources. The impact matrix, *D*_*α**i*_, describing the depletion of resource *α* by species *i* (Meszéna *et al.* 2006) is *D*_*α**i*_=*Q*_*α*_*a*_*α**i*_/*β*_*α*_, which substituted into the consumers equation, leads to

A 5

where  is the maximum growth rate and . Thus, the result is an effective interaction among the predators which is of LV type. It is customary to write equation (1.5) in terms of the carrying capacity *K*_*i*_, defined as the equilibrium population *N*_*i*_ attained in the absence of the other competitors, i.e. *K*_*i*_=*r*_*i*_/*C*_*i**i*_. In terms of it, equation (1.5) becomes identical to equation (1.1), with

A 6

Having a continuum *R*(*x*) of resources instead of a discrete set *R*_*α*_ does not introduce important difficulties. Simply, one should replace sums by integrals, replacing the coefficients of equation (1.5) by

A 7

A 8

A 9

One can also consider a continuum of species, labelled by their phenotypes *u*, so that equation (1.1) is replaced by equation (1.2) with *K*(*u*)=*r*(*u*)/*C*(*u*,*u*), *G*(*u*,*v*)=*C*(*u*,*v*)/*C*(*u*,*u*), and *r*(*u*) and *C*(*u*,*v*) given by obvious generalizations of equations (A 7) and (A 8).

It is clear that the presence in the kernel *G*_*i**j*_ of two different functions (compare with the most restrictive expression (1.1)) gives enough freedom to obtain a variety of kernel behaviours under different circumstances. A particularly popular choice is to assume that impact and sensitivity are proportional: *S*_*i**α*_=*ε**D*_*α**i*_, with a constant efficiency *ε*. In the continuum resource case the functions can be written in terms of a single utilization function *u*_*i*_(*x*) as *D*_*i*_(*x*)=*u*_*i*_(*x*) and *S*_*i*_(*x*)=*ε**u*_*i*_(*x*), leading to the classical expression (1.1). Slightly more general cases arise when the efficiency depends only on the resource, *ε*=*ε*_*α*_, or on the consumer, *ε*=*ε*_*i*_, or when dependence on the two types of species factorizes, *ε*=*v*_*i*_*w*_*α*_. In all these cases (if the efficiency is positive) one is led to a kernel *G*_*i**j*_, which is positive definite. In more general cases, one can have a kernel leading to instability of the coexistence state.

We conclude with two instances of ecological interactions in equation (A 3) which allows the stability to be tuned. First, a homogeneous discrete and infinite niche space in which all resources have the same internal dynamics *Q*_*α*_=*Q*, *β*_*α*_=*β*, ∀*α*, as well as the consumers: *d*_*i*_=*d*, ∀*i*. The interactions are taken to be

A 10

This models a situation in which the consumer *k* grows only by consuming its optimal resource *R*_*k*_, whereas it depletes also the neighbouring resources, *R*_*k*+1_ and *R*_*k*−1_. We have *r*_*i*_=*Q**g*, *K*_*i*_=*β*/*a*, *C*_*i**j*_=(*Q**g*/*β*)(*a**δ*_*i*,*j*_+*b*(*δ*_*i*,*j*−1_+*δ*_*i*,*j*+1_)), and *G*_*i**j*_=*δ*_*i**j*_+(*b*/*a*)(*δ*_*i*,*j*−1_+*δ*_*i*,*j*+1_) so that equation (1.1) is now

A 11

The natural solution, i.e. the one in which all species have positive non-zero population, is , ∀*i*. Its linear stability can be studied by linearization,  and substitution of the ansatz *δ**N*_*l*_≈*e*^*λ*_*q*_*t*^*e*^*i**q**l*^ (here ). We find , *q*∈[−*π*,*π*]. *λ*_*q*_ are the eigenvalues of −*C*_*i**j*_, and stability of  requires all these eigenvalues to be negative, i.e. *C*_*i**j*_ to be positive definite. When *a*>2*b*, then *λ*_*q*_<0 ∀*q*, and the natural coexistence solution is globally stable (see results in §2). It is unstable otherwise. In this example, there is no well-defined single utilization function and the positivity properties of the interaction kernel and thus the stability of the natural solution can be changed by varying the parameters.

As a second example, with non-constant carrying capacity, we consider a continuous distribution of resources and species on the line, and we take

which implies that the consumers of phenotype *u* grow only from the resource at location *x*=*u*, but they deplete a wider range characterized by *f*. This leads (in the continuous formalism) to *r*(*u*)=*s**Q**g*(*u*), *C*(*u*,*v*)=*s**Q**f*(*u*−*v*)/*β*, *K*(*u*)=*β**g*(*u*)/*f*(0) and *G*(*u*,*v*)=*f*(*u*−*v*)/*f*(0). In this example, by choosing the functions *g* and *f*, we can impose any desired combinations of carrying capacity and interaction kernel. Gaussianity or positive-definiteness are particular cases, no more natural in this generalization than alternative choices leading to non-positiveness, instability and thus exclusion zones between clumps of species.
